# RNA-Seq methods for imperfect samples: development, evaluation and applications

**DOI:** 10.1186/gb-2011-12-s1-p1

**Published:** 2011-09-19

**Authors:** Xian Adiconis, Lin Fan, David DeLuca, Andrey Sivachenko, Nathalie Pochet, Aaron Berlin, Sarah Young, Gad Getz, Aviv Regev, Chad Nusbaum, Andreas Gnirke, Joshua Z Levin

**Affiliations:** 1 Broad Institute of MIT and Harvard, Cambridge, MA 02141, USA

## 

Next-generation sequencing of RNA (RNA-Seq) is a powerful tool that can be applied to a wide range of biological questions. RNA-Seq provides insight at multiple levels into the transcription of the genome. It yields sequence, splicing and expression-level information, allowing the identification of novel transcripts and sequence alterations. We have been developing and comparing methods for samples that present a challenge: that is, those with low quantity and/or quality RNA.

RNA-Seq methods that start from total RNA and do not require the oligo(dT) purification of mRNA will be valuable for such challenging samples. Such methods use alternative approaches to reduce the fraction of sequencing reads derived from rRNA. We will present results from multiple approaches, including the use of not-so-random (NSR) primers for cDNA synthesis, low-C_0_*t* hybridization with a duplex-specific nuclease for light normalization and NuGEN’s Ovation RNA-Seq kit. We demonstrated that these three methods successfully reduce the fraction of rRNA to less than 13%, even when starting from degraded RNA. We compared the performance between these methods and with ‘gold standard’ RNA-Seq data (derived from samples with large quantities of high-quality RNA), using quantitative criteria that evaluate effectiveness for genome annotation, transcript discovery and expression profiling. The application of these methods to samples that contain degraded RNA and/or very low input amounts of RNA will also be presented.

